# Dorsal shaving affects concentrations of faecal cortisol metabolites in lactating golden hamsters

**DOI:** 10.1007/s00114-017-1536-7

**Published:** 2018-01-15

**Authors:** Sarah A. Ohrnberger, Katharina Brinkmann, Rupert Palme, Teresa G. Valencak

**Affiliations:** Institute of Physiology, Pathophysiology and Biophysics, Department of Biomedical Sciences, University of Veterinary Medicine Vienna, Veterinärplatz 1, 1210 Vienna, Austria

**Keywords:** Lactation, Heat dissipation, Shaving, Golden hamster, Glucocorticoids

## Abstract

Breeding of golden hamsters is classically performed at thermal conditions ranging from 20 to 24 °C. However, growing evidence suggests that lactating females suffer from heat stress. We hypothesised that shaving females dorsally to maximise heat dissipation may reduce stress during reproduction. We thus compared faecal cortisol metabolites (FCM) from shaved golden hamster mothers with those from unshaved controls. We observed significantly lower FCM levels in the shaved mothers (*F*_1,22_ = 8.69, *p* = 0.0075) pointing to lower stress due to ameliorated heat dissipation over the body surface. In addition, we observed 0.4 °C lower mean subcutaneous body temperatures in the shaved females, although this effect did not reach significance (*F*_1,22_ = 1.86, *p* = 0.18). Our results suggest that golden hamsters having body masses being more than four times that of laboratory mice provide a very interesting model to study aspects of lactation and heat production at the same time.

Golden hamsters (*Mesocricetus auratus*) are endemic to the Aleppinian plateau in Syria, where they were first caught and taken to the laboratory by researchers in 1932 (Aharoni 1932). From then on, their career manifested both as laboratory and as pet animals all over the world. Golden hamsters are tolerant towards high temperatures and have a remarkable reproductive potential. Their gestation period is the shortest known from placental mammals and only amounts to 16–18 days with very large litter sizes, ranging from 2 to 15 pups with a mean of 7.8 pups (Ohrnberger et al. 2016). Female hamsters produce substantial amounts of rich milk, leading to the rapid growth of their offspring from 3 to 5 g at birth to 30 g at weaning after a 19-day lactation period (Ohrnberger et al. 2016). Yet, this maternal energy investment into offspring growth comes at the cost of heat that passively warms up the body (Gamo et al. 2013b). Milk production, highly increased food intakes, and altered sleeping and foraging behaviour during lactation all contribute to the heat problem that female hamsters as well as other female mammals face when raising their young (reviewed in Speakman and Król 2005, 2011). In the past decade, a lot of research in laboratory rodents has been undertaken to elucidate the effects of increased internal heat production during lactation on female body temperature (Gamo et al. 2013a), milk production (Krol 2003), and offspring growth (Johnson and Speakman 2001; Johnson et al. 2001; Król and Speakman 2003; Valencak et al. 2013). Females are producing heat as a by-product of their increased metabolism (reviewed in Speakman and Król 2005, 2011). Thus, heating up during lactation may drive maximal energy intake in lactating females up to the point where energy intake can only be increased if females are relieved from the heat burden (Johnson and Speakman 2001). The easiest way to reduce the heat load is externally via lowering the ambient temperature conditions or by alleviating the dissipation of heat over the body surface, but females also have natural ways to do this, e.g., by actively seeking places away from the pups where they can cool down (Valencak et al. 2013).

Knowledge of the effects of the metabolic heat load on stress levels during lactation in a common but somewhat demanding laboratory rodent such as the golden hamster may help to raise awareness for this problem on a more general level and to implement amended breeding protocols in rodents and mammals in general. We measured faecal cortisol metabolites (FCM) noninvasively in lactating laboratory golden hamsters exposed to ad libitum feeding and raising naturally born (unmanipulated) litters to find out whether facilitated heat dissipation reduces stress in the mothers. We considered the quantification of FCM as a measure for distress in females as summarised (Palme 2012). Elucidating the role of stress on physiological limitations in nursing females has got a very large potential applicability for breeding mammals both in the farming and the laboratory animal industry.

Female mice are experimentally shaved dorsally to prevent overheating, and this turned out to be an efficient measure to improve their energy intake and milk output (Król et al. 2007). Does shaving also affect FCM levels during lactation? Golden hamsters are substantially bigger than mice and thus have altered heat loss over the body surface. Shaving females may be a simple, cheap and quickly reversible measure to facilitate heat dissipation, but it clearly requires approval by local Animal Welfare and Ethical Review Bodies (AWERBs) before it can be applied to improve breeding success in females. Experimental data just as ours may therefore help to find out if shaving females would facilitate loss of excessively produced metabolic heat caused by lactation. Long-term elevated stress levels are known to affect the whole organism starting from increased blood glucose concentrations, altered behavioural patterns, and inhibited growth up until affecting fertility and modulating the immune system (Romero and Butler 2007).

We obtained faecal samples to quantify faecal cortisol metabolites from lactating golden hamster mothers split into two groups: (i) from females that had their fur shaved dorsally and (ii) golden hamster mothers with intact fur. Measuring FCM enabled us to assess adrenocortical activity noninvasively as done previously and reviewed in Palme (2012). Our study used FCM protocols previously validated for golden hamsters by applying an ACTH challenge test (Chelini et al. 2010). We chose to sample during the time when female energy demands are highest and most likely heat load was maximal for the mothers. Thus, we collected the faecal samples on days 8 and 9 of lactation when juvenile hamsters still rely on maternal milk but have grown bigger to demand more milk than at parturition. We hypothesised that shaved mothers have lower FCM levels due to the improved dissipation of heat over the body surface and thus reduced heat stress. We did not address a potential correlation between reproduction and adrenocortical activity as this question was already addressed in Chelini et al. (2011) who showed that indeed golden hamster females not becoming pregnant after the pairing had higher FCMs than those that conceived successfully (Fig. 2 in Chelini et al. 2011).

## Materials and methods

### Animals, housing and hygiene conditions

Laboratory golden hamsters were obtained from Charles River Laboratories, (Sulzfeld, Germany) with specific pathogen-free status. These animals were used to start a breeding stock. A total of 15 females were used in this study. The animals were between 61 and 334 days old, were regularly paired, and allowed to raise litters consecutively. We housed them individually in polycarbonate cages (Eurostandard Type IV, 595 × 380 × 200 mm, Techniplast, Italy). Cages were equipped with autoclaved wood shavings (Abedd, Austria) and were changed once a week, unless it coincided with the day of parturition, when females were not disturbed. Equally, the cages of late pregnant and early lactating (− 12 until 24 h postpartum) females were not opened for cleaning or measurements of body temperature but rather were left undisturbed. All animals were kept on an L:D 16:8 photoperiod at 22° ± 2 °C. Females were paired with males for 4 days, after which the males were removed to avoid aggressive interactions between the animals. All animals had ad libitum access to food and water throughout the experiment. They received commercial hamster diet V 2144 (Ssniff, Soest, Germany) during the experiment. The room where the experiment took place is isolated from outside and was accessible only to 4 people, following strict hygiene protocols: No animals other than with specific pathogen-free status were brought in once taken out, and animals were never taken back into the facility again to maintain the high hygiene status. Twice per year, the hygiene status of the colony is determined according to FELASA recommendations by AnLab (Prague, Czech Republic) and can be released upon request to the lead author.

### Shaving

On day 6 of lactation (DOL 6), all females from the shaved subgroup were shaved dorsally using a Wella Contura Hair Clipper (Wella, Germany). Females were briefly taken out of their cages, separated carefully from the pups, and were gently but firmly held and shaved dorsally. Less than 3–4 min later, they were returned to the nest. Because hamsters are used to being handled (weighed) daily, they immediately started nursing again; therefore, we were confident that our shaving procedure itself caused no additional stress for the animals. To match the disturbance in the two groups, the unshaved females were likewise taken out of the nest, were handled for ca. 3–4 min (without shaving but with the clipping device on and making the noise) and then carefully returned to the nest.

### Subcutaneous body temperature

At the age of 8 weeks, we implanted subcutaneous transmitters in all 15 females, which allowed daily measurements of subcutaneous body temperature (IPTT-300, BioMedic Data Systems®). Our used device is factory calibrated for a temperature range between 32–43 °C (details can be found under: http://www.bmds.com/products/transponders/iptt-300/specs). The female was briefly taken out of the cage, held between the shoulders, and the transmitter was carefully implanted with a syringe in the subcutaneous tissue over the animal’s lumbar vertebrae. This allowed us not only to identify the animal even when it was co-housed with the male, but also to obtain recordings of subcutaneous body temperature with the help of a hand-held reading device (DAS-7006/7s, BioMedic Data Systems®). The transmitter was located dorsally and not close to the mammary glands to avoid any effects by the suckling pups on skin temperature. No anaesthesia was needed for implantation of the transmitters, as the procedure usually takes less than 3 min, and females are used to being handled.

### Data collection

From DOL 1 until weaning (DOL 19), we continuously measured mothers’ body masses and subcutaneous body temperatures. All measurements were taken daily between 08:00 am and 11:00 am to ensure reproducibility. The day when pups were observed for the first time was defined as the day of parturition, which was referred to as day 0 of lactation. Please note that we paired the same set of 15 females three times to maximise statistical power. No female in our study conceived more than twice and if a female did not get pregnant for one of the three repeats, she was defined as non-reproductive in this particular round (*n* = 2 females). The non-reproductive group consisted of shaved and unshaved females before reproduction and two females that did not breed during the course of our study. Eight females raised two litters consecutively, so the same individual females were raising one litter being shaved and another litter with intact fur. Four females only got pregnant once. One extreme outlier from the unshaved group had to be excluded from the dataset mostly likely due to a measurement error.

Faecal samples therefore were collected when mothers were lactating in a shaved, in an unshaved or in a reproductively inactive state to evaluate individual baseline FCM levels.

### Collection of faecal samples

**Fig. 1:**
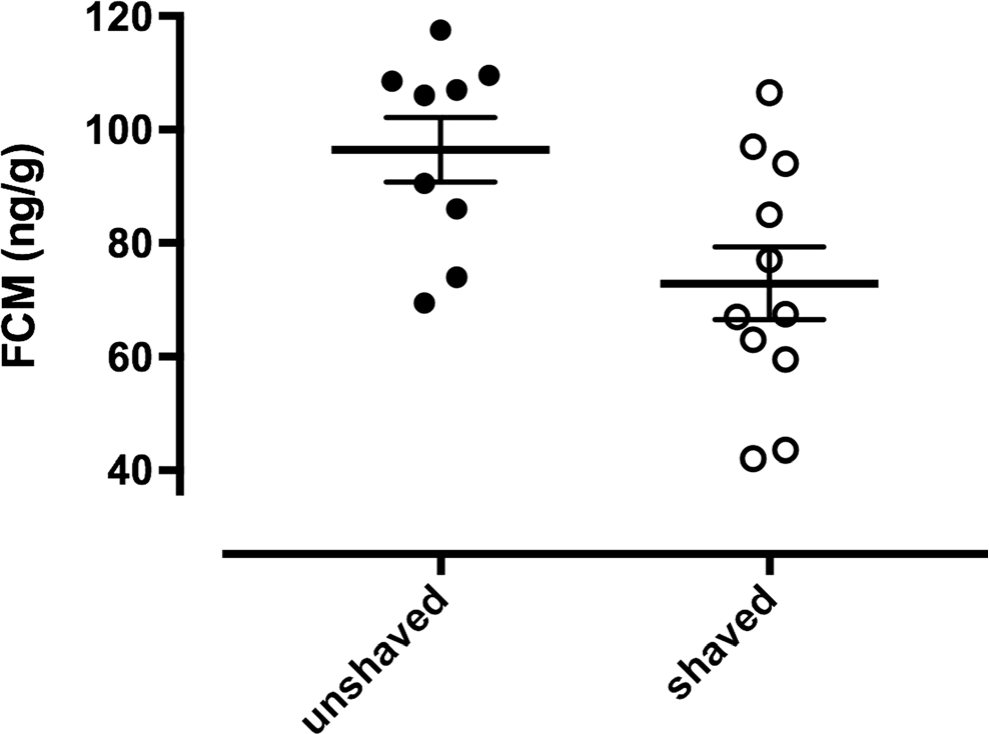
Faecal cortisol metabolites (FCM; mean ± sem) in lactating unshaved (*n* = 9, full circle) and shaved (*n* = 11; open circle) female golden hamsters

Faecal samples of shaved and unshaved lactating females were collected on 2 days, DOL 8 and DOL 9 (2 days after shaving, 10 days before weaning), respectively. Sample collection for all three groups (shaved, unshaved, non-reproductive) always took place between 09:00 and 12:00 am when hamsters usually are at rest. Collecting faecal samples took less than 2 min. After the fresh faecal pellets were identified by visual inspection of the bedding and the humidity state of the faecal pellets, the home cage of each female was briefly opened and the samples were obtained directly from the cage floor by using a forceps without disturbing the litter. Dry faeces pellets have a lighter colour and are less moist as freshly defecated ones. Faecal samples of the female could easily be distinguished from faecal pellets of the litter by their difference in size. Faecal samples were then stored at − 20 °C until assayed. For a total of eight females, we were able to collect faecal samples on all the three occasions the other seven females were sampled only once or twice (see above and in Fig. 1).

### Steroid analysis

Faecal steroids were extracted using the methanol-based procedure outlined in Palme et al. (2013). Lyophilisation was not necessary due to the consistency of golden hamster faeces. After homogenisation of each faecal sample (by using a mortar), 50 mg were weighed and stored at − 20 °C until assayed. Each faecal sample was shaken on a vortex (Velp Scientifica Vortex mixer, USA) at 20 rpm with 1 ml of 80% methanol and subsequently on a thermomixer (Eppendorf Thermomixer compact 5350, Eppendorf, Germany) at 500 rpm for 15 min. The suspension was then centrifuged at 2500×*g* for 5 min (Eppendorf centrifuge 5424, Eppendorf, Germany). Faecal cortisol metabolites were quantified in an aliquot of the extract (50 μl further diluted 1:10 with assay buffer) using a group specific 11-oxoaetiocholanolone enzyme immunoassay (EIA measuring glucocorticoid metabolites with a 5β-3α-ol-11-one structure) successfully validated for the golden hamster (Chelini et al. 2010). Details of this assay were first described by Möstl et al. (2002). Concentrations of FCM are expressed as nanograms per gram faecal dry matter.

### Statistical analyses

All statistical analyses were run using R, version 3.2.0 (R Core Team 2015). We applied multifactorial linear mixed effect models by using the R package lme4 (Bates et al. 2015). Models therefore always included mother identity as random factor as two litters were born from the same mothers. We also included mothers’ body mass, age and parity as covariates. Significance level was set at *p* = 0.05, means and standard errors of the means are provided for shaved (*n* = 11), unshaved (*n* = 9) and non-reproductive females (*n* = 13).

The full dataset is readily available upon request to sarah.ohrnberger@vetmeduni.ac.at.

## Results

Shaved golden hamster mothers had significantly lower FCM levels than unshaved ones (*F*_1,22_ = 8.69, *p* = 0.0075, Fig. 1). To rule out a potential effect of individual body mass on FCM concentrations, we included mass into our linear mixed effects model with random factor female and found no partial effect whatsoever on FCM (*F*_1,22_ = 0.0067, *p* = 0.9355). There were also no significant partial effects of mothers’ age (*F*_1,22_ = 0.116, *p* = 0.737) and parity (F_1,22_ = 0.459, *p* = 0.5051) on FCM concentrations.

Mean weaned litter size in our study was 8.3 ± 1.2 pups and 7.7 ± 1.3 pups in shaved (*n* = 11) and unshaved (*n* = 9) females, respectively. There was no significant difference in weaning weights between young raised by shaved or unshaved mothers (*F*_1,18_ = 0.27, *p* = 0.61) with 274.6 ± 22.7 g and 271.6 ± 45.9 g, respectively. There was however a significant partial effect of litter size on FCM (*F*_1,22_ = 5.55, *p* = 0.028) with females having intermediate litter sizes of 5–7 showing higher FCMs than those raising litters of 8–11 pups.

Non-reproductive and unshaved lactating females had FCM concentrations that were not significantly different from each other and amounted to 104.53 ± 10.9 and 94.5 ± 13.5, respectively (*F*_1,22_ = 0.52, *p* = 0.48).

Unshaved and shaved lactating females had mean subcutaneous body temperatures of 37.3 ± 0.9 °C and 36.9 ± 0.8 °C, respectively (*F*_1,22_ = 1.86, *p* = 0.1826, Fig. 2). Lactating golden hamsters generally had higher subcutaneous body temperatures than non-reproductive females (*F*_1,20_ = 24.07, *p* = 0.0001). There was no significant effect of mothers’ age, parity, individual body mass and litter size on subcutaneous body temperature (all *p* > 0.4).

**Fig. 2:**
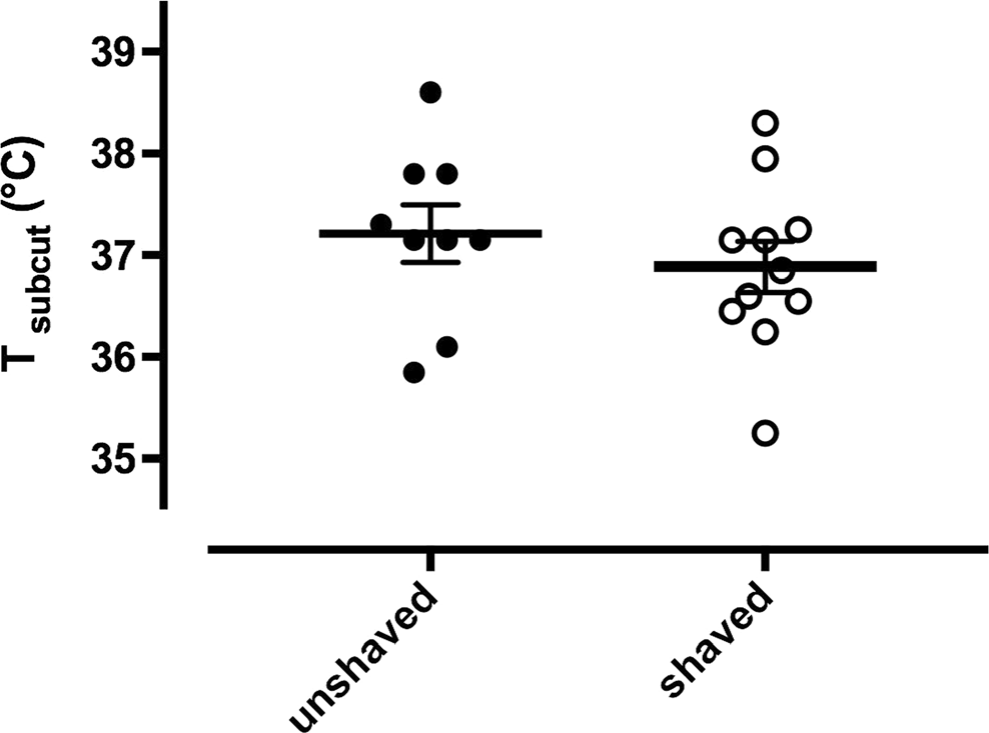
Subcutaneous body temperature (Tsubcut; mean ± sem) in lactating unshaved (*n* = 9; full circle) and shaved (*n* = 11; open circle) female golden hamsters

## Discussion

### Lower stress in shaved lactating females

Our study shows that lactating unshaved golden hamsters had 0.4 °C higher subcutaneous body temperatures than shaved hamsters although the effect did not reach significance in our study presumably caused by the small effect and sample sizes. Dorsal shaving seemingly relieved lactating females from some heat load, resulting in lower FCM levels than in golden hamster mothers with intact fur. Thus we conclude that shaved mothers had lower adrenocortical activity. Previously, FCM levels in golden hamsters were quantified during pair formation, mating and pregnancy (Chelini et al. 2011). Interestingly, the concentrations presented in Chelini et al. (2011) were within a similar range as our data in the unshaved females presented here. In the same study, non-reproductive golden hamsters were found to have higher FCM levels than pregnant females in the first week of gestation (Chelini et al. 2011). Not gestating or even not conceiving may therefore lead to higher FCM values than observed in lactation. Please note that golden hamsters are solitary mammals and usually do not tolerate conspecifics or even the male in their cage so we speculate that females failing to conceive may actually be suboptimal as reference or control due to their possibly higher general adrenocortical activity, which would not be related to the process of lactation as such. The process of lactation brings about so many metabolic changes that it may be oversimplified to easily extrapolate FCM values from lactating females to those individuals that do not become pregnant and served as non-reproductive references.

Maximised energy metabolism (Speakman and McQueenie 1996; Speakman 2000), which generally is observed in lactating females, gives rise to maximal heat production and heat stress. Impressively, the heat load seems to be constant over lactation, i.e., the duration of full 3 weeks (Ohrnberger et al. unpublished results).

Heat production may have the largest effects on FCM on DOL 8 and 9 when we collected our faecal samples. At that time, all juveniles still fed on milk only but yet have grown to a bigger size than in the first week of lactation. Also 8–9 days old pups suckle significantly more than on the first day after parturition (Ohrnberger SA, pers. observation) so pup demand on maternal milk production is likely to be highest around DOL 10 when pups start opening their eyes, eating up pelleted food and being independent from maternal thermoregulation. Indeed, we found significantly higher FCM concentrations in females with intact fur compared to the shaved mothers. As we found previously that DOLs 8–13 clearly represent the energetically most challenging phase in lactation for the hamsters (Ohrnberger et al. 2016), we consider our finding as biologically most relevant. Notably, we observed that females raising mediocre litter sizes (5–7 pups) had the highest FCMs not the ones raising large litters of 8–11 pups. There are several possible explanations for this finding and we feel that future observations on FCM values in lactation need to address the relationship between individual maternal heat load, litter size and milk production, which clearly is most relevant parameter in large litters. One explanation that we would like to bring up is that empirically, only few females in a population are able to nurse large litters and probably therefore it is that female cohort that shows increased heat dissipation, or even more suppressed non-shivering thermogenesis and also lower FCMs that produce the most milk. This is plausible in the light of previous work done on this question (Reznick et al. 2000). Alternatively, mothers raising large litters of 8–11 pups have lower FCM values than mothers of 5–7 pups because their pups grow slower (Ohrnberger SA pers. observation), move less, and cause fewer stress for the mothers due to their lower general activity on DOLs 8 and 9 when we sampled the faeces.

During lactation, all organ systems work at exceptionally high rates and each female ingests up to three times more food and energy than a reproductively inactive female at baseline (Ohrnberger et al. 2016). Intestinal processes generally, and the postprandial increase of resting metabolism specifically, boost heat production together with the energy expenses for milk synthesis (Speakman and Król 2005). Not astonishing therefore, thermoregulatory processes such as the activity of uncoupling proteins are largely shut down during lactation as shown in mice (*Mus musculus*) (Trayhurn et al. 1982; Król et al. 2011).

To corroborate the importance of the timing, one has to argue that until ca. DOL 12 the pups live entirely from milk but yet at the same time they have higher energy demands than around the time of birth (Ohrnberger et al. 2016). From ca. DOL12 onwards, golden hamster pups supplement their milk intake with solid food and may compensate for lower milk intake by ingesting larger quantities of pelleted food (Ohrnberger et al. 2016).

In golden hamsters, our intervention of shaving the female alleviated the heat burden by 0.4 °C and reduced stress in females. Our finding that the heat load in unshaved lactating hamsters manifests in higher adrenocortical activity is novel, and we predict that it may give rise to future studies in other model systems. We feel that our data are unsuitable to contribute to the general discussion whether and to what extent reproduction affects stress in females in general (Paul et al. 2010) as we focused on the heat stress response in two experimental groups differing in the thickness of the fur only.

Please note that while we measured subcutaneous body temperatures, Gamo et al. (2013b) showed that in lactating laboratory mice core body temperatures increased by almost 2 °C. We therefore might have failed to find a significant effect of the shaving intervention on body temperature because our transmitters were implanted subcutaneously and therefore provided less effect size than intraperitoneally implanted device as used in Gamo et al. (2013b). Overheating may indeed prove a major limiting factor for milk production in mammals in general. Newly, we demonstrate here that overheating may also affect stress levels in nursing females, which may be of great interest in terms of animal welfare. In a recent, parallel study in lactating golden hamsters, we found that at 30 °C, golden hamsters significantly reduced their litter size compared to females kept at 22° and 5° (Ohrnberger et al. 2016). These litter size reductions culminated during the middle part of the lactation period due to low energy intake and negligible milk production at temperature conditions of 30 °C (Ohrnberger et al. 2016). Future studies may therefore help elucidating intrinsic physiological limitations of milk production by more carefully considering stress and female well-being during lactation.

### The effects of facilitated heat dissipation in other model organisms

Our observation highlights the importance of all sorts of measures alleviating heat dissipation to the environment. This was previously done by exposing mothers to temperatures below their thermoneutral zone in mice (Johnson and Speakman 2001), in Mongolian gerbils (*Meriones unguiculatus*) (Yang et al. 2013), in Siberian hamsters (*Phodopus sungorus*) (Paul et al. 2010) to enumerate the rodent examples and even in lagomorphs (Valencak et al. 2010). In none of these experiments FCM were quantified, but all studies found an increase in milk production caused by reduced ambient temperatures and improved heat dissipation. Alternative to lowering ambient temperature conditions, shaving female mice and offering them chilled drinking water helped to increase milk production and juvenile growth rate (Marai et al. 2001; Król et al. 2007; Valencak et al. 2013). Many authors argue that animals kept in the laboratory should be held at thermo-neutral conditions, ranging around 30 °C for most laboratory rodents (Hylander and Repasky 2016; Helppi et al. 2016). Careful re-considerations on the temperature requirements of lactating females therefore may improve the well-being of the mothers in accordance of the 3 R’s in laboratory animal research (Russell and Burch 1959). We propose that special attention to the thermal environment of the females is given so that even though their body temperatures are consistently higher (Gamo et al. 2013b) and their endogenous heat production is lowered (Król et al. 2011), they still have some flexibility for behavioural measures in order to getting rid of excess metabolic heat. Valencak et al. (2013) showed previously that female MF1 mice readily switch cages to pick up chilled water as well as food and that this again increased milk production. Favourable breeding conditions for laboratory rodents therefore should involve means to release heat to the environment, while at the same time allowing warm and insulated nests for the growth of the young. Notably, there seem to be totally diverging needs for both pups and females in lactation: while pups grow faster and bigger when kept at temperatures above room temperature due to the lower energy costs spent on thermoregulation, their mothers only have impaired scope of metabolic action at higher temperatures due to own hyperthermia and often abandon or cannibalise their litter (Ohrnberger et al. 2016).

### Conclusion

During lactation, when metabolism achieves manifold higher turnover rates over non-breeding conditions, female golden hamsters have 0.4 °C lower subcutaneous body temperatures (Fig. 2) and reduced FCM values when shaved dorsally (Fig. 1). Our study sheds light on the female’s heat stress during lactation, especially in the “elite” golden hamster mothers, which combine a very short gestation time with large litters and a quick and swift nursing period of 18 days. This reproductive strategy may exacerbate individual maternal heat load by maximising energy turnover in the females. While shaving alone will not be enough to efficiently lower stress in lactating females, we are proposing that shaving may contribute to facilitate heat dissipation in lactating females when lowering ambient temperatures is not possible. Shaving lactating golden hamster females in our study may thus have reduced their heat burden caused by lactation, and this in itself may reduce “stress” as reflected in lower FCM levels (Fig. 1). The golden hamster is known for its solitary behaviour and this, in combination with the outstanding reproductive potential and its body mass being more than four times that of a laboratory mouse with all the consequences on heat dissipation and thermoregulation, suggests that it provides a very interesting model where to study aspects of lactation and heat production.
